# Maintenance of subsynaptic myonuclei number is not driven by neural input

**DOI:** 10.3389/fphys.2023.1266950

**Published:** 2023-09-26

**Authors:** Lloyd P. Ruiz, Peter C. Macpherson, Susan V. Brooks

**Affiliations:** ^1^ Department of Molecular and Integrative Physiology, University of Michigan, Ann Arbor, MI, United States; ^2^ Department of Biomedical Engineering, University of Michigan, Ann Arbor, MI, United States

**Keywords:** muscle, myonuclei, subsynaptic, innervation, neuromuscular junction, aging

## Abstract

The development and maintenance of neuromuscular junctions (NMJ) are supported by a specialized population of myonuclei that are referred to as the subsynaptic myonuclei (SSM). The relationship between the number of SSM and the integrity of the NMJ as well as the impact of a loss of innervation on SSM remain unclear. This study aimed to clarify these associations by simultaneously analyzing SSM counts and NMJ innervation status in three distinct mouse models of acute and chronic NMJ disruption. SSM were identified using fluorescent immunohistochemistry for Nesprin1 expression, which is highly enriched in SSM, along with anatomical location beneath the muscle fiber motor endplate. Acute denervation, induced by surgical nerve transection, did not affect SSM number after 7 days. Additionally, no significant changes in SSM number were observed during normal aging or in mice with chronic oxidative stress (*Sod1* ^−/−^). Both aging WT mice and *Sod1* ^−/−^ mice accumulated degenerating and denervated NMJ in skeletal muscle, but there was no correlation between innervation status of a given NMJ and SSM number in aged or *Sod1* ^−/−^ mice. These findings challenge the notion that a loss of SSM is a primary driver of NMJ degradation and leave open questions of the mechanisms that regulate SSM number as well as the physiological significance of the precise SSM number. Further investigations are required to define other properties of the SSM, such as transcriptional profiles and structural integrity, to better understand their role in NMJ maintenance.

## Introduction

Maintenance of the neuromuscular junction (NMJ), the synapse between a motor neuron and muscle fiber, is supported in part by a specialized population of muscle fiber nuclei known as subsynaptic myonuclei (SSM). SSM are located subjacent to the muscle endplate and are responsible for the local enrichment of acetylcholine receptors (AChRs) and other proteins required to establish NMJ structure and function (Apel et al., 2000; Sanes and Lichtman, 2001; Schaeffer et al., 2001; Li et al., 2011; Shi et al., 2012). The specialization of the SSM involves both activity- and load-dependent mechanisms (Purves and Sakmann, 1974a; Purves and Sakmann, 1974b; Ruegg, 2005; Haddix et al., 2018; Cisterna et al., 2020) and likely trophic support from the associated motor neuron (Saini et al., 2021). Thus, disruption of the integrity of an NMJ would be expected to impact anterograde signaling and the structure, function, or number of SSM, while alterations in SSM structure, number or function may also signal back to trigger degenerative changes at the NMJs. The impact of an acute loss of innervation on SSM has not been defined nor has the role of the loss of SSM on NMJ degeneration been fully elucidated.

Degeneration of NMJs and loss of innervation are often cited as important contributors to the age-associated loss of muscle referred to as sarcopenia (Hashizume et al., 1988; Doherty et al., 1993; Delbono et al., 2021). The hypothesis of a causative link between decreased integrity of the NMJ and sarcopenia is based largely on reports of an accumulation of NMJs that display morphological abnormalities including partial or complete loss of overlap of pre- and post-synaptic structures and discontinuous staining pattern of postsynaptic AChRs during aging (Valdez et al., 2010; Jang and Van Remmen, 2011; Li et al., 2011; Willadt et al., 2016; Deschenes et al., 2022; Verma et al., 2022).

Recent studies of the relationship between NMJ degeneration and SSM number have produced conflicting results. While Liu et al. (2017) found a reduction in SSM number in old animals compared to adults, a recent report by Ang et al. (2022) concluded that age-associated differences in SSM number are modest and may be secondary to NMJ structural modifications. Furthermore, Larouche et al. (2021) found no differences in baseline SSM numbers between young and old mice; however, an increase in perisynaptic nuclei 28-days after sciatic nerve transection appeared in young mice only. In contrast to the findings of Larouche et al. (2021), Bai et al. (2022) reported a decrease in synaptic nuclei number 16 days after denervation, with the decreased number remaining stable out to 30 days. Finally, Bai et al. (2022) also found that the number of SSM in mouse model of amyotrophic lateral sclerosis (SOD1^G93A^) was less than that of its wildtype counterpart 90-days after birth, suggesting a relationship between innervation and SSM number. These collective findings indicate that questions of whether loss of innervation triggers loss of SSM or whether loss of SSM triggers degenerative changes at NMJs are in need of further investigation.

The goal of the present study was to further clarify the association between SSM number and NMJ integrity. We addressed this goal through simultaneous analysis of counts of total perisynaptic myonuclei and specific SSM along with assessments of NMJ innervation status using identical methods across models of both acute and chronic NMJ disruption. SSM were identified using an antibody specific to Nesprin1. Nuclear envelope spectrin repeat proteins (Nesprins) are highly expressed in skeletal muscle (Grady et al., 2005; Duong et al., 2014) and act to anchor myonuclei at the NMJ (Grady et al., 2005; Packard et al., 2015; Holt et al., 2019; Holt et al., 2019). The nesprin-1- alpha-2 isoform is specifically expressed in the synaptic nuclei in adult muscle fibers (Holt et al., 2019). A novel aspect of the present study was the use of Nesprin1 staining in conjunction with markers for pre- and post-synaptic structures to establish associations of SSM with innervation status.

Based on the known importance of the motor neuron in SSM specialization (Ruegg, 2005; Saini et al., 2021) along with reports of fewer SSM at NMJs in muscle fibers of old compared with adult animals (Liu et al., 2017) and links between SSM number and NMJ structural modifications (Ang et al., 2022; Bai et al., 2022), our overall hypothesis was that a relationship exists between the loss of innervation and a loss of SSM or *vice versa*, i.e., a loss of SSM drives disruption of NMJ structure. To address this hypothesis, we determined SSM number in three models of NMJ degeneration in mice. First, we asked whether acute denervation induced by surgical nerve transection triggered a loss of SSM. We next examined muscles from adult and old wild type mice to establish whether SSM number changed during normal aging. Lastly, we studied muscles from mice deficient in the antioxidant enzyme superoxide dismutase 1 (*Sod1* ^−/−^ mice). *Sod1* ^−/−^ mice develop degenerative changes at NMJs during adulthood, including fragmentation of the endplates and frank muscle fiber denervation (Ivannikov and Van Remmen, 2015; Deepa et al., 2019).

We tested the specific hypotheses that the number of Nesprin1 positive nuclei sub-adjacent to muscle fiber endplates would be lower 1) in muscles following nerve transection compared with innervated controls, 2) for muscles of old compared with adult wild type mice, and 3) for muscles of *Sod1* ^−/−^ compared with wild type mice. We further hypothesized that in all cases, the loss of nesprin positive myonuclei would be correlated with the extent of NMJ disruption, from fully innervated through to fully denervated.

## Material and methods

### Animals

Male and female mice of the C57BL/6 background and male and female *Sod1* ^−/−^ mice and wild type (WT) littermates were used in this study. All mice were housed in specific-pathogen-free conditions, with ad-libitum access to standard laboratory food and water and were subjected to a 12- hour light/dark cycle. The animal experiments described here were reviewed and approved by the University of Michigan Institutional Animal Care and Use Committee (IACUC).

### Sciatic nerve transection

C57BL/6 mice approximately 6-months of age were subjected to sciatic nerve transection (SNT) and randomly assigned to either a 3-day or 7-day recovery group. The procedure was as follows. First, mice were anesthetized with 5% isoflurane as required to ensure no response to tactile stimuli, followed by continuous administration of 2% isoflurane for maintenance. Next, carprofen (5 mg/kg) was administered as a preoperative analgesic. The hindlimbs were carefully shaved and the skin was cleaned with chlorhexidine and 70% alcohol. A small skin incision (<10 mm) was made 1 mm posterior and parallel to the femur, and the superficial biceps femoris was split to expose the sciatic nerve. The sciatic nerve was then transected, and a 5 mm segment was removed. The incision was closed with wound clips (Autoclip, BD Clay Adams, Franklin Lakes, NJ). The same procedure was performed on the contralateral leg. After surgery, the mice were placed on a heating pad and closely observed until they recovered from the anesthetic. Mice recovered for three or 7 days before being sacrificed.

### Tissue collection

For tissue collection, mice were anesthetized with intraperitoneal injection of avertin (0.5 mg/g). The bilateral gastrocnemius (GTN) muscles were carefully dissected from mice exposed to SNT (N = 3/time-point, 2 males and 1 female at each timepoint), as well as from 6-month-old young WT mice, 28-month-old aged WT mice, 6-month-old *Sod1* ^−/−^ mice, and 12-month-old adult *Sod1* ^−/−^ mice (N = 5/group, 3 males and 2 females). Muscles were briefly fixed for 10 min in 4% paraformaldehyde for immunohistochemical analysis. Tissue was then cryoprotected in a 20% sucrose solution, then snap-frozen in embedding medium O.C.T compound (Fisher Scientific) with liquid nitrogen cooled isopentane to maintain the integrity of the membranes. The tissue samples were stored at −80°C until thawed and used for analysis. Following removal of the tissues, deeply anesthetized mice are euthanized by an overdose of anesthetic followed be the creation of a pneumothorax.

### Immunohistochemistry

Myofiber bundles were mechanically teased from GTN muscles under a dissecting microscope and processed for immunohistochemistry. The myofiber bundles were first blocked for 1 h at room temperature with a solution of 5% goat serum (Gibco), 1% bovine serum albumin (Fisher Scientific), 0.5% Triton X-100 (Sigma-Aldrich), and 0.01% sodium-azide (Sigma-Aldrich), then blocked for 30 min with 10% Mouse IgG, Fab fragment (Jackson ImmunoResearch). The myofiber bundles were then incubated overnight at 4°C with primary antibodies (Nesprin1 rabbit-polyclonal antibody (Invitrogen) at 1:500 dilution and Neurofilament (2H3)/Synaptic vesicle glycoprotein 2A mouse-monoclonal antibody (NF/SV2) (2H3 1:50 and SV2 1:25 dilutions). 2H3 was deposited to the DSHB by Jessell, T.M./Dodd, J and SV2 was deposited by Buckley, K.M. (DSHB Hybridoma Products 2H3 and SV2). The following morning, bundles were washed with phosphate-buffered saline (PBS), treated with secondary antibodies (goat-anti-rabbit 594 at 1:500 dilution and goat-anti-mouse 647 at 1:1000 dilution) (Invitrogen), and co-stained with alpha-bungarotoxin (α- BTX) conjugates 488 (Invitrogen) at 1:1000 dilution and DAPI (4′,6-diamidino-2-phenylindole, dihydrochloride) (Invitrogen) at 1:1000 dilution for 1 h at room temperature. The myofiber bundles were then washed with PBS, mounted onto slides with DAKO fluorescence mounting media, and imaged using oil immersion and Nikon A1 High-sensitivity confocal microscopy.

**FIGURE 1:**
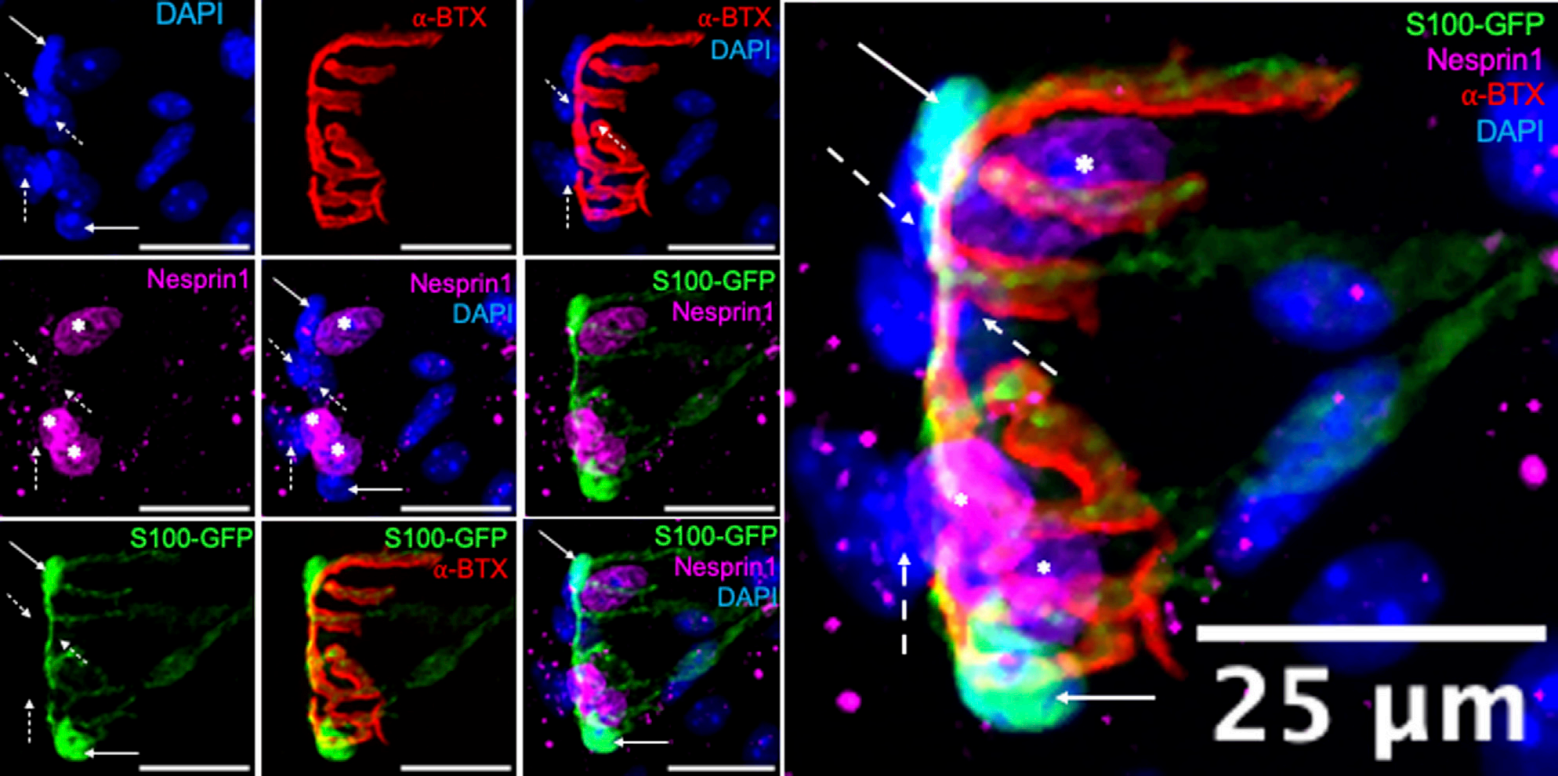
*Terminal Schwann cells do not express Nesprin1*. Immunofluorescence of 6-month-old S100-GFP gastrocnemius fibers with terminal Schwann cells labeled in (green), nuclei (blue), acetylcholine receptors (AChR, red), and Nesprin1 (magenta), Solid arrows indicate terminal Schwann cells that do not express Nesprin1, dashed line arrows indicate synaptic nuclei that do not 360 express Nesprin1, and asterisks indicate SSM, those that express Nesprin1 and overlap with AChRs. Images on the left are individual or partially merged channels. Image on right is enlarged merged image of all individual channels.

To analyze and quantify perisynaptic nuclei and subsynaptic myonuclei (SSM) and assess innervation status, Z-stack confocal images were processed and projected as 3-D images in ImageJ as described (Schneider et al., 2001). For the purposes of the present manuscript all images are presented as 2-D maximum intensity projections. We performed a preliminary analysis to confirm that the expression of Nesprin1, a protein involved in the organization and anchoring of the nucleus to the cytoskeleton, would differentiate between SSM and terminal Schwann cell nuclei associated with the NMJ. This analysis of Nesprin1 expression was performed in adult S100-GFP mice. In the S100- GFP animal model, terminal Schwann cells are labeled with GFP under the control of the S100 promoter, whereas myonuclei do not express GFP (Figure 1). Nesprin1, labeled in magenta, was entirely absent from terminal Schwann cell nuclei, as indicated by the solid arrows, highlighting that Nesprin1 expression represents a feature of SSM that allows for its use to distinguish SSM from perisynaptic terminal Schwann cell nuclei at the neuromuscular junction (Figure 1). Furthermore, Nespirn-1+/GFP- nuclei that did not overlap with the endplate were also observed. These nuclei were not counted as SSM and therefore not included in our analysis, but represent an interesting population of nuclei for future study. Finally, our analysis also revealed synaptic nuclei, indicated by dashed line arrows, that do not express GFP or Nesprin1. These observations emphasize the importance of rigorous scrutiny when examining SSM and underscores the relevance of using both anatomical location and Nesprin1 expression as our identifying criteria for SSM based on the high likelihood of miscounting SSM when relying entirely on proximity to the endplate.

In the analysis of all images to quantify SSM and innervation status, the nerve and presynaptic terminal were labeled by NF/SV2 and postsynaptic acetylcholine receptors were stained with α-BTX. Endplates were classified as fully innervated when more than 80% of the NF/SV2 signal overlapped with the α-BTX signal, partially denervated when the overlap was between 10% and 80%, and denervated when the overlap was less than 10%. Perisynaptic myonuclei were defined as nuclei showing overlap with the α-BTX signal, while Nesprin1 protein expression served as a marker for SSM. A nucleus was classified as a SSM by expression of Nesprin1 along with at least 25% overlap of the DAPI signal with α-BTX.

### Data presentation and statistics

A minimum of 15 NMJs were analyzed per individual muscle in each mouse. The data are presented as means ± standard error of the mean with data points for individual muscles included on each figure. Statistical analysis was performed by two-tailed unpaired Student’s t-test, and one-way ANOVA where applicable. Significance was set *a priori* at *p* < 0.05. Only a *p* value of less than 0.05 is labeled in the figure.

## Results

### Acute nerve injury does not elicit a change in subsynaptic myonuclei number out to 7 days

**FIGURE 2:**
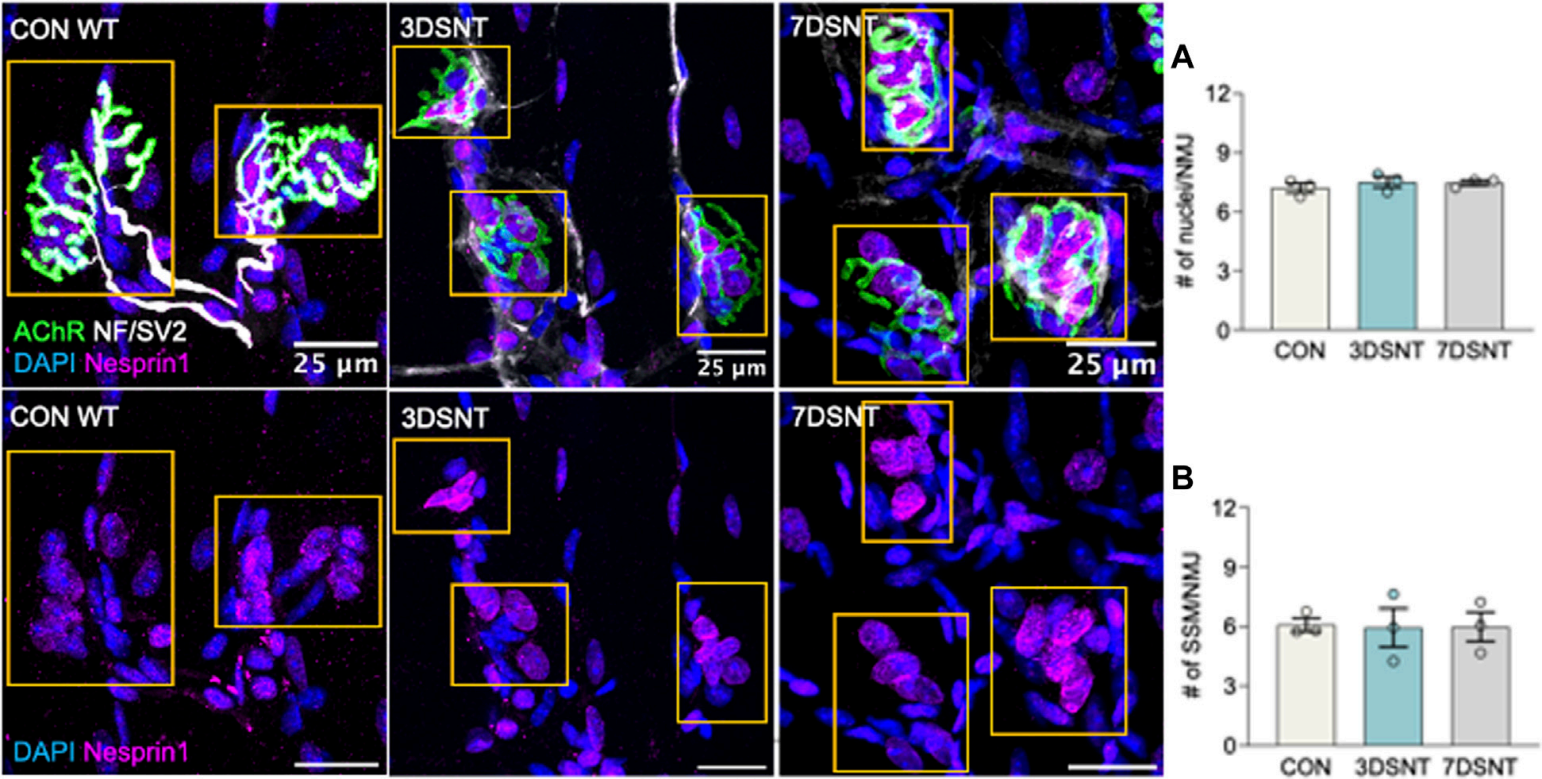
Acute denervation does not alter synaptic nuclei number. Upper panel: representative immunofluorescent images of gastrocnemius muscle fibers for control (CON) muscles and muscles 3 days and 7 days after sciatic nerve transection (3DSNT and 7DSNT) from 6-month-old WT mice stained for DAPI (blue), AChRs (green), NF/SV2 (white), and Nesprin1 (magenta). Lower panel: same images without AChR and NF/SV2 to highlight Nesprin1 nuclear staining. Region of interest (ROIs) highlight the endplate and are indicated by dashed boxes (yellow). Scale bars = 25 µm. **(A)** Quantification average number perisynaptic and **(B)** subsynaptic nuclei number per NMJ. Individual data points represent average values for each muscle sample. Bars represent mean values with error bars ±SEM (n = 3/group, 15 NMJs/sample). There were no significant differences in the average numbers of perisynaptic nuclei/NMJ or in subsynaptic myonuclei number in control, 3DSNT, and 7DSNT.

The effect of acute denervation on SSM was quantified in myofiber bundles from GTN muscles 3 (3DSNT) and 7 days (7DSNT) following SNT by immunohistochemistry as described above. Uninjured control mice displayed fully innervated endplates with pretzel-like morphology, with perisynaptic and subsynaptic nuclei labeled by DAPI and Nesprin1 (Figure 2 upper panel images). Three days after injury, the nerve began to clear and a loss of innervation was evidenced by the remains of only a faint NF/SV2 signal, and by 7 days after injury, NF/SV2 staining is completely absent (Figure 2 upper panel images). When assessed by 3-D image analysis, we identified no differences in the average number of perisynaptic nuclei per endplate between control (x = 7.2 ± 0.2 nuclei), 3DSNT (x = 7.5 ± 0.2 nuclei), and 7DSNT (x = 7.5 nuclei ±0.2). We also found no change from control levels (x = 6.0 ± 0.2 nuclei) (Figure 2A) in subsynaptic myonuclei (SSM) number 3 days or 7 days post-SNT (3DSNT: x = 5.0 ± 0.2 nuclei; 7DSNT: x = 5.5 nuclei ±0.197) (Figure 2B). Our results indicate that acute denervation does not significantly impact the number of SSM at the NMJ.

### Subsynaptic myonuclei number is not influenced by NMJ degeneration associated with normal aging or chronic oxidative stress

**FIGURE 3:**
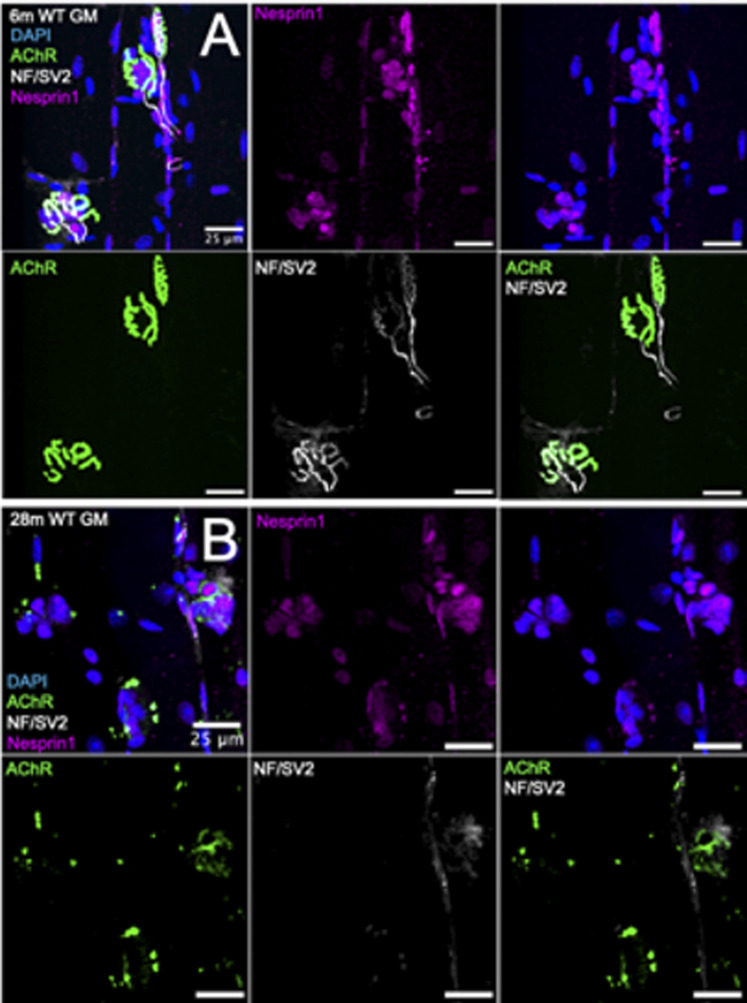
Visualization of synaptic myonuclei in young and old wild type mice. Immunofluorescent images of gastrocnemius muscle fibers from **(A)** 6-month **(B)** 28-month wild type mice and stained for DAPI (blue), AChR (green), NF/SV2 (white), and Nesprin1 (magenta). Scale bars = 25 µm.

**FIGURE 4:**
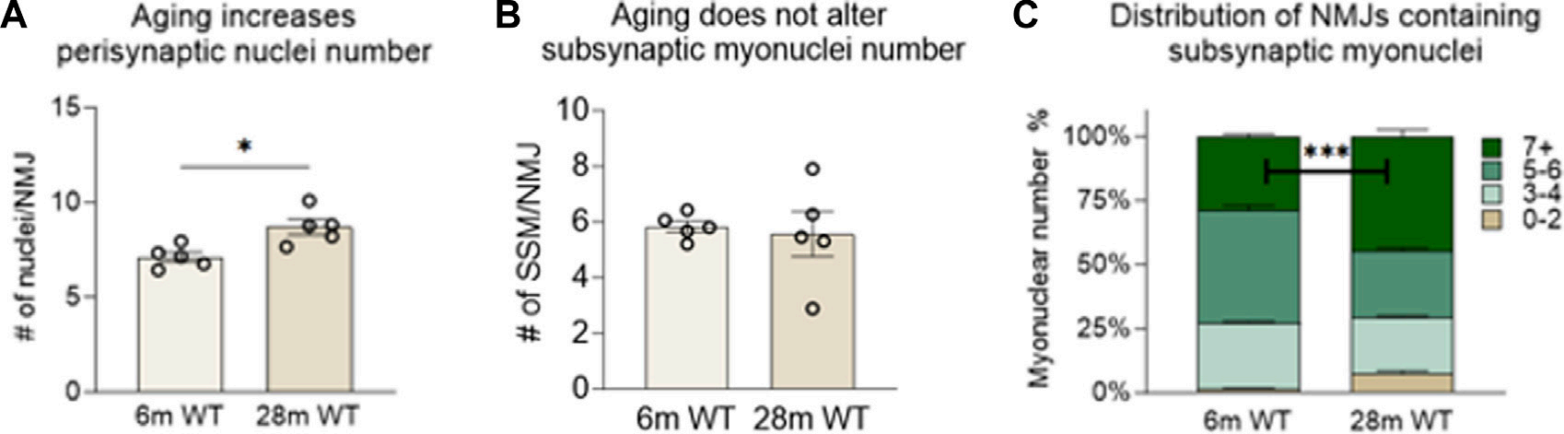
Number of subsynaptic myonuclei remains unchanged by age. Average number of **(A)** perisynaptic nuclei and **(B)** subsynaptic nuclei per NMJ. Individual data points represent Average value or each muscle sample. Bars represent mean values with error bars ±SEM (n = 5/group, 15 NMJs/sample). **(C)** Data on subsynaptic myonuclear number expressed as a percentage of The NMJ Containing 0–2 nuclei (tan bars), 3-4 nuclei (light green bars), 5-6 nuclei (green bars) or 7 or more nuclei (dark green bars). ***p < 0.0001). Bars represent mean values with error bars ±SEM.

**FIGURE 5:**
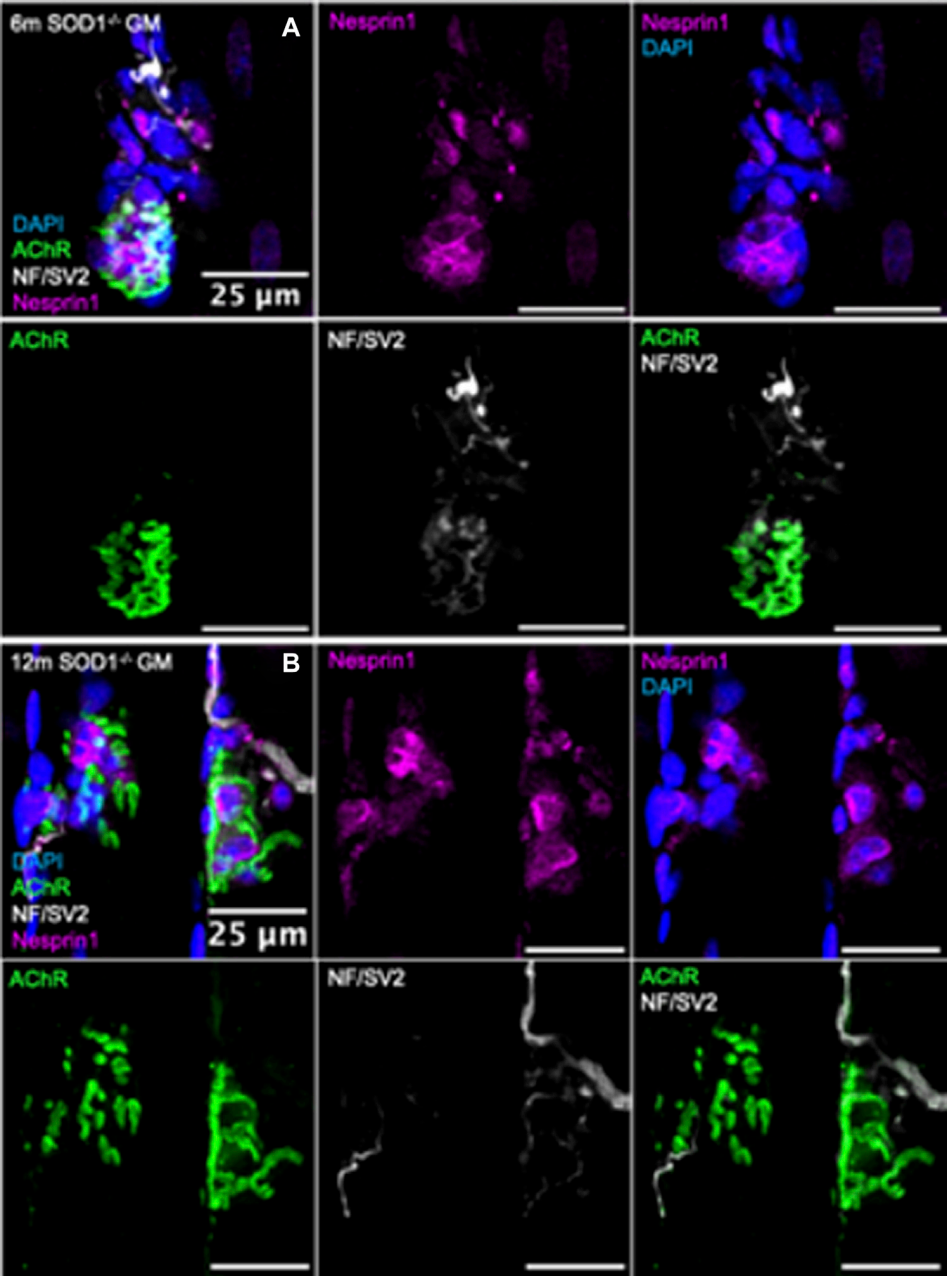
*Visualization of synaptic myonuclei in mice deficient in Sod1* ^*−/−*^ *.* Immunofluorescent images of gastrocnemius muscle fibers from **(A)** 6-month and **(B)** 12-month *Sod1* ^*−/−*^ mice stained for DAPI (blue), AChR (green), NF/SV2 (white), and Nesprin1 (magenta). Scale bars = 25 µm.

**FIGURE 6:**
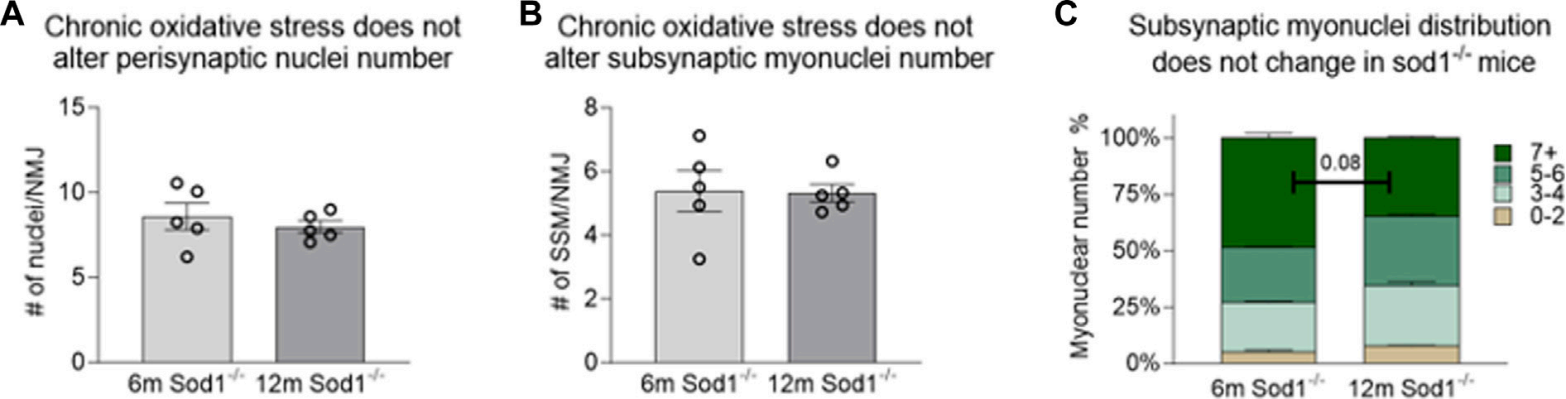
*Number of subsynaptic myonuclei remains unchanged by Sod1 deletion.* Average number of **(A)** perisynaptic nuclei and **(B)** subynaptic myonuclei per NMJ. Individual data points represent average value for each muscle sample. Bars represent mean values with error bars ±SEM (n = 5/group, 15 NMJs/sample). **(C)** Data on subsynaptic myonuclear number expressed as a percentage of the NMJ containing 0–2 nuclei (tan bars), 3-4 nuclei (light green bars), 5-6 nuclei (green bars) or 7 or more nuclei (dark green bars). Bars represent mean values with error bars ±SEM.

To investigate the effect of chronic NMJ disruption on SSM number and the relationship of innervation status at the NMJ to SSM, we analyzed isolated myofiber bundles from GTN muscles of 6- and 28-month-old WT mice (Figure 3) and 6- and 12-month-old Sod1^−/−^ mice (Figure 5). In WT mice, we identified an approximately 20% increase in the number of perisynaptic nuclei per endplate for muscles of 28-month compared with 6-month mice (Figure 4A) (*p* < .01), but when SSM specifically were compared we found no differences in SSM number between 6-month and 28-month mouse endplates (Figure 4B). We then reanalyzed and further scrutinized the data by generating histograms as similarly presented in (Liu et al., 2017) with the number of NMJs containing specific numbers of SSM counted. Bin sizes for the histograms were 0–2, 3–4, 5-6 or 7+ SSM per NMJ (Figure 4C). While this analysis revealed a greater representation in muscles of 28-month-old compared with 6-month-old mice of NMJs with 0–2 nuclei (7% vs 1%), there was simultaneously a greater proportion at 28 months of NMJs with 7 or more SSM, 45% and 29% for 28-month and 6-month mice, respectively. There were no differences between the age groups for the percentage of endplates containing 3-4 SSM or 5-6 SSM (Figure 4C).

Similar analyses were performed on NMJs from muscle fiber bundles of 6- and 12-month *Sod1* ^−/−^ mice. The average number of SSM were similar between 6-month *Sod1* ^−/−^ mice and age-matched WT mice (Figure 4B; Figure 5B). There was also no change between 6 and 12 months in *Sod1* ^−/−^ mice in the average number of perisynaptic or SSM per NMJ (Figures 6A,B); however, in the case of Sod1 deletion and contrary to the findings in aging WT mice, we observed a trend (*p* = 0.08) toward fewer NMJs with high numbers (7+) of SSM at the older age (Figure 6C) (6-month *Sod1* ^−/−^: 49% and 12-month *Sod1* ^−/−^:35%). This decrease in the percentage NMJs with 7+ SSM was accompanied by similarly higher numbers of NMJs with either 3-4 or 5-6 SSM, but neither increased reached statistical significance.

### Subsynaptic myonuclei number is not influenced by innervation status

**FIGURE 7:**
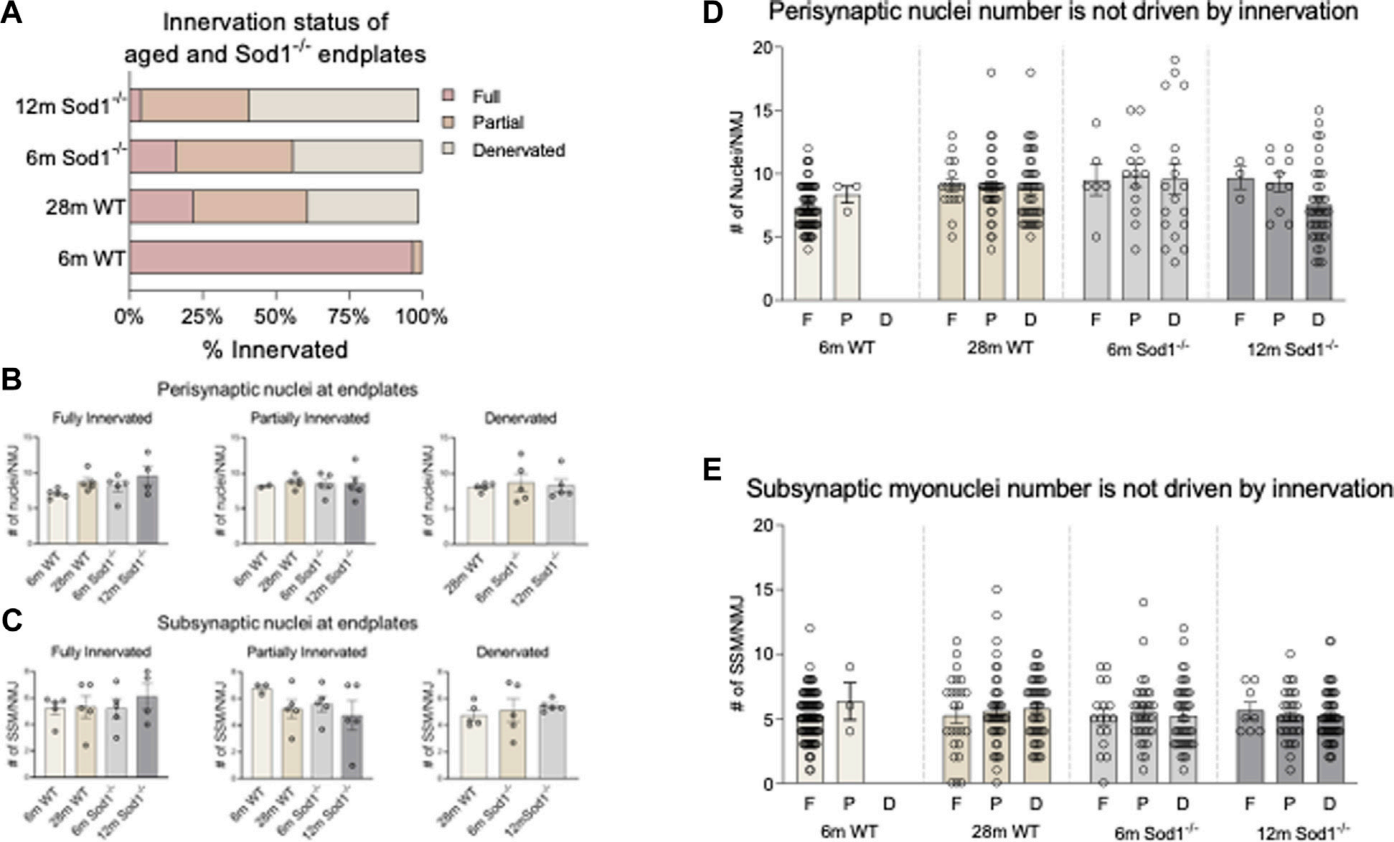
*Synaptic nuclei number is not driven by innervation status*. **(A)** Distribution of innervation status of young and aged wildtype and Sod1^−/−^ mouse endplates. Endplates were classified as fully innervated if NF/SV2 signal overlapped with more than 80% of the endplate, partially denervated if overlap was <80–10%, and fully denervated if overlap was less than 10%. **(B)** Average number of perisynaptic nuclei at fully innervated, partially innervated and denervated wildtype and Sod1^−/−^ mice. **(C)** Average number of subsynaptic nuclei at fully innervated, partially innervated and denervated wildtype and Sod1^−/−^ mice. **(D)** Number of perisynaptic nuclei at an NMJ based on innervation status. Each point represents the number of perisynaptic nuclei at an endplate. F, Full; P, Partial; D, Denervated. The total number of NMJs analyzed are as follows 6 m WT F = 88, *p* = 3, D = 0; 28 m WT F = 25, *p* = 42, D = 48; 6 m Sod1^−/−^ F = 17, *p* = 34, D = 42; 12 m Sod1^−/−^ F = 8, *p* = 33, D = 52. **(E)** Number of subsynaptic nuclei at an NMJ based on innervation status. Each point represents the number of subsynaptic nuclei at an endplate. F, Full; P, Partial; D, Denervated.

To investigate whether the number of SSM is influenced by the innervation state of a given NMJ during aging or with exposure to chronic oxidative stress, we grouped SSM numbers based on NMJ innervation status (Figure 7). Consistent with previous reports (Deepa et al., 2019) we found the majority of endplates in GTN muscles of 6-month WT mice to be fully innervated, with a very small percentage being partially denervated. In contrast, in 28-month WT mice, only 22% of endplates are fully innervated, 39% partially denervated and 38% were identified as denervated. In GTN muscles of 6-month Sod1^−/−^ mice, more than 84% of the endplates were denervated or partially denervated, and by 12 months of age, there were essentially no fully innervated endplates remaining in Sod1^−/−^ mice (Figure 7A). Despite these dramatic shifts in innervation status between genotypes and across age, we observed no differences in the number of SSM at a given NMJ (Figure 7B; Figure 7C). We also analyzed the number of nuclei present at every individual NMJ imaged and in 6-month WT mice, we did not observe any denervated endplates (Figures 7D,E). While we observed increased variability in nuclei counts under conditions of significant NMJ denervation, in aged WT mice and with deletion of Sod1, the cause of this variability remains unknown. Our results demonstrate that SSM number is not affected by the innervation status in aging WT or *Sod1* ^−/−^ mice.

## Discussion

Based on the knowledge that neural activity is an important regulator of the specialization of SSM (Purves and Sakmann, 1974a; Haddix et al., 2018; Cisterna et al., 2020)we hypothesized that a strong relationship would exist between NMJ innervation and the distribution of myonuclei at and around the synapse. Contrary to our hypotheses, we observed no effect of acute denervation on the number of SSM nor any differences in SSM number between fully innervated, partially innervated, and fully denervated endplates in two different models of progressive NMJ degeneration. In addition to the rigor in these data provided by our simultaneous analysis of multiple muscle fiber denervating conditions, we have also, for the first time, used both anatomical position of nuclei as well as a marker reported to be specific for SSM (Grady et al., 2005; Holt et al., 2019) in our identification of different populations of myonuclei and demonstrated that location is not sufficient to discriminate SSM from other nuclei proximal to the NMJ. These findings are significant based on disparate reports in the literature that have evaluated individual models separately using purely anatomical definitions of SSM.

Our studies of sciatic nerve transections allowed us to directly test for the impact of both electrical activity at the endplate and anterograde signaling from the motor neuron on SSM number. Our observation of no significant changes from innervated controls in the number of perisynaptic nuclei or Nesprin1+ SSM at 3 days or 7 days post-injury is contrary to reports of a decrease in SSM number 5 days after sciatic nerve axotomy (Picchiarelli et al., 2019). Although these data reported 5 days after denervation are inconsistent with our findings at 3 and 7 days, we cannot rule out the possibility of transient changes in the localization of nuclei occur due to migration following denervation. In contrast to the report of Picchiarelli et al. (2019) of a decrease in SSM following denervation, Larouche et al. (2021) attributed an increase in perisynaptic nuclei 28 days following nerve transection to muscle stem cell engraftment at the NMJ as a mechanism to restore the population of SSM following denervation. Again, while inconsistent with our findings, we may very well have observed a similar increase in synaptic nuclei at this later time point. Physiological injury to the muscle fiber membrane triggers a response in which nuclei migrate towards the injury site within 24 h, initiating a repair process that is independent of satellite cells (Roman et al., 2021). The number of nuclei are reported to subsequently show a significant reduction back toward the pre-injury state by 48 h (Roman et al., 2021). These findings suggest that muscle nuclei, perhaps due at least in part to their association with muscle membranes, are highly responsive to muscle and nerve injury and can initiate rapid migratory behavior.

The possibility that nuclear positioning is dynamic and responsive to neural input and/or muscle injury is interesting when considered with our observation of an apparent upregulation of Nesprin1 in muscle nuclei after injury. In *drosophila*, Nesprin1 has been shown to serve as a track upon which RNA is transported upon to regulate synaptic growth and functionality during development (Packard et al., 2015). Following nerve injury, tracks may be formed to transport trophic factors towards the synapse and promote stability of the endplate. Alternatively, it is possible that nerve injury may cause temporary migration of SSM to initiate a degradation process of the NMJ following nerve injury, deep bulk RNA sequencing of sciatic nerve samples reveals the activation of an acute immune response which remains active for up to 14 days. (Yi et al., 2015). Additionally, single-nuclei RNA sequencing showcased substantial changes in transcription profiles in myonuclei classified type IIb, which were further divided into subpopulation of type IIb1 and type IIb2 due to the dynamic nature of myonuclei transcription and the significant impact of denervation after nerve injury (Lin et al., 2022). Collectively, characterization of numerous properties of myonuclei strongly support the notion that, even if they are not lost, the emergence of local domains within myofibers likely has a major impact on the responses to denervation (Bruusgaard and Gundersen, 2008).

Our findings of no changes in the number of SSM in GTN muscles of aged (28-months) compared with young (6-months) WT mice are contrary to those of previous studies that reported fewer SSM in the tibialis anterior and diaphragm muscles of 24-month-old compared to 6-month old mice (Liu et al., 2017). Moreover, Ang et al. (2022) recently reported a modest reduction in the number of perisynaptic myonuclei in both male and female mice between 6- and 28-months of age, but the specific identity of the nuclei counted remains unknown due to the presence of alternative cells such as terminal Schwann cells and immune cells at the synapse (Alhindi et al., 2021; Rios et al., 2021). Histograms meant to help visualize the likelihood of specific numbers of SSMs present at a given NMJ have been previously used to establish the optimal number of nuclei required to maintain the synaptic domain and to provide insight into the functional impact of a loss of several nuclei (Liu et al., 2017). Even upon analysis of our data in the manner used by (Liu et al., 2017), our observation of an increase in the number of NMJs presenting with high numbers of SSM in muscles of old mice is contrary to their findings. Further, nearly 50% of the NMJs in muscles of 6- month *Sod1* ^*−/−*^ mice, which are phenotypically similar to aged WT mice (Deepa et al., 2019), displayed 7 or more SSM. Finally, our observation of a simultaneous increases in muscles of 28- compared with 6-month-old mice in the proportion NMJs with 0–2 nuclei and NMJs with 7+ SSM precluded any general conclusion about an overall increase or decrease with aging in the number of SSM at the NMJ. The numbers of SSM reported in the literature have shown a high degree of variability, likely based largely on the different criteria used among studies for identification of SSM (Liu et al., 2017; Picchiarelli et al., 2019; Ang et al., 2022; Bai et al., 2022). While some studies classify SMM as those with >25% DAPI signal covered by alpha-bungarotoxin, others use a more stringent definition requiring >70% of the nuclear volume to be overlapping with alpha-bungarotoxin. In the present study, we utilized Nesprin1 as a marker of SSM (Grady et al., 2005; Packard et al., 2015; Holt et al., 2019), in addition to overlap with the endplate to identify SSM and avoid the possibility of misidentification of terminal Schwann cells that are integrated into the NMJ as SSM. Our findings of a significant increase in the number of perisynaptic nuclei defined anatomically in muscles of 28- month compared with 6-month WT mice but no difference between the groups when Nesprin1 staining was included as a criterion for classification of SSM strongly supports the possibility that using physical location of myonuclei and their proximity to alpha-bungarotoxin staining is insufficient to identify SSM in immunofluorescent images, even when confocal microscopy is used to generate 3D reconstructions. The results of the present study, using the rigorous criteria of both overlap with acetylcholine receptors and Nesprin1 staining to identify SSM, support the conclusion that the number of SSM remains stable across the lifespan under normal physiological conditions and with the presence of chronic oxidative stress.

The etiology of sarcopenia, a progressive age-associated loss of skeletal muscle mass and function, remains incompletely understood. Evidence suggests that alterations during aging in the structure of the synaptic apparatus and a decrease in the degree of muscle fiber innervation may contribute to sarcopenia (Valdez et al., 2010; Jang and Remmen, 2011; Li et al., 2011; Willadt et al., 2016; Deschenes et al., 2022). Although recent studies postulate that SSM, which provide support to the synaptic apparatus, may be linked to the integrity of the NMJ (Santos et al., 2020; Saini et al., 2021) our findings of no change with aging in either WT mice or Sod1^−/−^ mice, a model of accelerated neuromuscular aging (Deepa et al., 2019), in SSM indicates that any contribution of alterations in SSM to breakdown of the NMJ and loss of innervation is not related to SSM number. This conclusion is further supported by our analysis of the relationship between the state of innervation of a given endplate and the associated number of SSM. Although our data on the extent of denervation during normal aging and with Sod1 deficiency aligned well with previous literature, in which muscles of *Sod1* ^−/−^ mice showed a similar distribution of innervated endplates at 12 months of age as observed in WT mice at 28 months (Deepa et al., 2019; Pollock et al., 2023), we observed no substantial differences based on innervation status in the number of SSM nuclei present. These observations showing a lack of any relationship between innervation status and SSM number were contrary to the recent report of Ang et al. (2022) who concluded that any changes with aging in SSM number were likely secondary to NMJ structural modifications. Interestingly, we observed a higher degree of variability in SSM number in the muscles of mice subjected to sciatic nerve transection and in Sod1^−/−^ mice compared with muscles of young WT mice. The degree of variability was comparable to the variance in synaptic nuclei number previously reported in Sod1^−/−^ mice (Bai et al., 2022) and in 4EBP1-transgenic mice (Ang et al., 2022). An increase in variability is perhaps not surprising based on the heterogeneity of the state and duration of denervation or reinnervation across NMJs. Together these findings suggest that the position of nuclei may not necessarily be static, rather that migration of myonuclei and other cell types is ongoing in response to changes in innervation status or other signals that may trigger changes in both position and function of nuclei as well as NMJ disruption. A dynamic population of nuclei may explain some of the disparate findings in the literature. It is important to note that our study has limitations, such as examining only two time points for each genotype, which are unlikely to capture transient changes in myonuclei number that may occur throughout the mouse lifespan, particularly in response to injury as observed by Bai and colleagues. In addition, although using Nesprin1 as one of the identifying features of SSM is a strength of the study, the mechanisms regulating Nesprin1 expression are not known. The hypothesis that neural input is at least in part responsible for maintaining Nesprin1 expression is not unreasonable. In fact, since many of the NMJs in the muscles of 12-month *Sod1* ^−/−^ mice have likely been chronically denervated, our observation of a decrease in the percentage of NMJs with high numbers (7+) of SSM in this group is consistent with a loss of Nesprin1 expression with “long” term loss of neural input. Future studies defining the regulation of Nesprin1 expression are warranted.

In addition, in this study we did not control for variations in muscle fiber type which introduces intriguing complexities related to the NMJ. Specifically, within the extensor digitorum longus (EDL) muscle of the rat, a notable difference exists between NMJs associated with Type 1 fibers, which have been documented to be morphologically larger, compared to those linked to Type 2 fibers. In contrast, in the soleus muscles, the observation is reversed, with Type 2 fibers exhibiting larger NMJ morphologies. (Deschenes et al., 2013). More recent studies in mice suggest a correlation wherein fast-twitch fibers are more prone to innervation by smaller endplates, while slow twitch fibers exhibit larger post-synaptic structures, as documented by Mech et al. (2020). However, when considering the context of aging, recent findings support the prevailing notion that aging primarily affects fast-twitch fibers. Instead, the latest evidence suggests that the impact of aging is more likely to be muscle-specific rather than solely determined by fiber type, as indicated by Skinner et al. (2021). Nonetheless, it is worth noting that the gastrocnemius muscle of mice contains only 5% Type 1 fibers (Augusto et al., 2004) indicating that we have evaluated NMJs from almost exclusively Type 2 Fibers. Further investigation is warranted to delve into the quantity and properties of subsynaptic myonuclei in various contexts. Finally, we have not characterized other properties of the nuclei, such as shape (Battey et al., 2022; Kalukula et al., 2022; Rader and Baker, 2022), expression of nuclear proteins, chromatin structure, or transcriptional differences. Despite these weaknesses, our findings clearly challenge the notion that a loss of SSM is a primary driver of NMJ degradation and suggest that changes in the number of SSM may not imbue significant physiological repercussions to the endplate.

Based on our findings and existing literature, we conclude that maintaining the NMJ is not solely dependent on the number of SSM present, but also on other factors including the transcriptional profile of pre and postsynaptic nuclei (Kim et al., 2020; Perez et al., 2021). Age-related alterations to nuclei and their products may also contribute to the degradation of the NMJ (Yousefzadeh et al., 2021). While our study does not show acute changes in SSM number following nerve injury, future studies should investigate transient and chronic changes in myonuclei number after nerve insult. Additionally, identifying DNA damage in SSM and isolating Nesprin1 positive nuclei for single nuclei sequencing could provide insights into age-related changes to the transcriptional landscape of these nuclei and how motor neuron innervation defines their transcriptional profile (Roman et al., 2021; Bai et al., 2022).

## Data Availability

The raw data supporting the conclusion of this article will be made available by the authors, without undue reservation.
